# Negative differential resistance and bias-modulated metal-to-insulator transition in zigzag C_2_N-*h*2D nanoribbon

**DOI:** 10.1038/srep43922

**Published:** 2017-04-06

**Authors:** Jing-Jing He, Yan-Dong Guo, Xiao-Hong Yan

**Affiliations:** 1 College of Electronic Science and Engineering, Nanjing University of Posts and Telecommunications, Nanjing 210046, China; 2 Key Laboratory of Radio Frequency and Micro-Nano Electronics of Jiangsu Province, Nanjing 210023, Jiangsu, China; 3 College of Science, Nanjing University of Aeronautics and Astronautics, Nanjing 210016, China

## Abstract

Motivated by the fabrication of layered two-dimensional material C_2_N-*h*2D [*Nat. Commun.* 6, 6486 (2015)], we cut the single-layer C_2_N-*h*2D into a zigzag nanoribbon and perform a theoretical study. The results indicate that the band structure changes from semiconducting to metallic and a negative differential resistance effect occurs in the *I*-*V* curve. Interestingly, the current can be reduced to zero and this insulator-like state can be maintained as the bias increases. We find this unique property is originated from a peculiar band morphology, with only two subbands appearing around the Fermi level while others being far away. Furthermore the width and symmetry of the zigzag C_2_N-*h*2D nanoribbon can be used to tune the transport properties, such as cut-off bias and the maximum current. We also explore the electron transport property of an aperiodic model composed of two nanoribbons with different widths and obtain the same conclusion. This mechanism can be extended to other systems, e.g., hybrid BCN nanoribbons. Our discoveries suggest that the zigzag C_2_N-*h*2D nanoribbon has great potential in nanoelectronics applications.

Two-dimensional (2D) materials have attracted tremendous attention for applications in future nanoelectronics owing to their physical, electrical and chemical properties^1^. As a typical 2D material, graphene exhibits high strength, extraordinary conductivity and excellent transmittance^2^,^3^. However, the absence of a band gap limits its application in nanoelectronics and optoelectronics^4^,^5^,^6^,^7^. Recently, a novel 2D layered material C_2_N-*h*2D has been synthesized via a simple wet-chemical reaction^8^,^9^. The C_2_N-*h*2D material possesses evenly distributed nitrogen atoms and holes. Compared to graphene, its band opens a direct gap of 1.70 eV, and the field-effect-transistor fabricated thereon has a high on/off ratio of 10^7^, suggesting great potential in device application. In previous studies on fullerences, carbon nanotubes and graphene-based structures, researchers have found the negative differential resistance (NDR) effect which can be applied to logic operators, molecular switch, memory, oscillator, etc refs 10, 11, 12, 13, 14. For example, Ren *et al*.^14^ have reported NDR occurs in a doped armchair graphene nanoribbon-based junction, such NDR behavior derives from the interaction between the narrow density of states of the doped leads and the discrete states in the scattering region. Do *et al*.^15^ have found the NDR effect may be severely affected by the roughness of ribbon edges in a *p*^+^/*p* junction based on zigzag-edged graphene. Khoo *et al*.^16^ have observed NDR phenomenon in carbon chain-nanotube junctions and find that it is caused by an energy misalignment in even-chain model, however it is produced by the asymmetric distortions of the conducting resonances in odd-chain model. In these theoretical work, all structures are molecular junctions. In this paper, we present a periodic nanoribbon (zigzag C_2_N-*h*2D)-based model, and investigate the effect of width and symmetry on its electronic and transport properties. By performing first-principles calculations, we find C_2_N-*h*2D changes from semiconducting to metallic after been cut into a nanoribbon. In order to explore the physical mechanism, we first set up a periodic model. A NDR effect appears in this periodic system, surprisingly, the current can reduce to zero and then this state can be maintained as the bias increases. In a few nano-sized systems, this kind of phenomenon is rarely reported. In-depth study shows that this unique phenomenon stems from the peculiar band morphology where there are only two subbands near the Fermi level and others are far away. In addition, the width and symmetry of the zigzag C_2_N-*h*2D nanoribbon can tune the cut-off bias and the maximum current. We also show that similar electron transport occurs in a heterogeneous model and other materials. These transport properties are quite useful for future nanoelectronic device applications.

## Results

**Figure 1:**
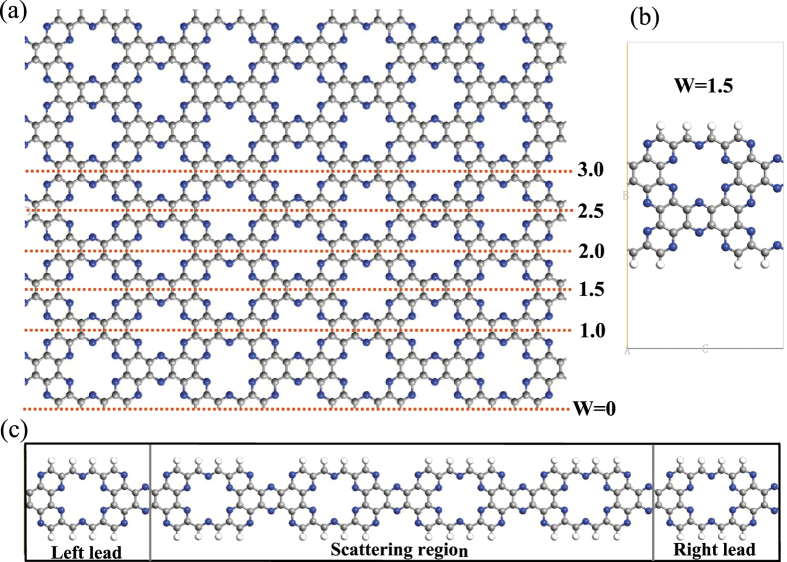
Schematic illustration of (**a**) Zigzag C_2_N-*h*2D structures with different widths (W = 1.0, 1.5, 2.0, 2.5 and 3.0); (**b**) The unit cell of W = 1.5 models. (**c**) The two-probe system of zigzag C_2_N-*h*2D nanoribbon (W = 1.0). Nitrogen, carbon and hydrogen atoms are shown by blue, gray and white balls, respectively.

Compared to graphene, there are more ways to cut the single-layer C_2_N-*h*2D into a zigzag nanoribbon due to the presence of holes and nitrogen atoms. Classification can be carried out according to the atomic species of nanoribbon edges, e.g., carbon and nitrogen-edged or carbon-edged. In our research, we select the structure whose edge atoms are all carbon atoms. To show the nanoribbons more intuitively and clearly, here we use W to denote the width and choose W = 1.0 to 3.0 models to study. The schematic illustration of zigzag C_2_N-*h*2D nanoribbons with different widths is shown in Fig. 1(a). The unit cell of W = 1.5 model is chosen to be shown in Fig. 1(b). Obviously, when W is an integer (W = 1.0, 2.0 and 3.0), the structure is symmetric. If the W is a non-integer (W = 1.5 and 2.5), the structure has no symmetry. The two-probe configuration could be divided into three parts, the left semi-infinite zigzag C_2_N-*h*2D nanoribbon electrode, the central scattering region and the right semi-finite zigzag C_2_N-*h*2D nanoribbon electrode (as shown in Fig. 1(c)), the length of the central scattering region is 59 Å which is enough to avoid direct coupling between left and right electrodes^17^.

**Figure 2:**
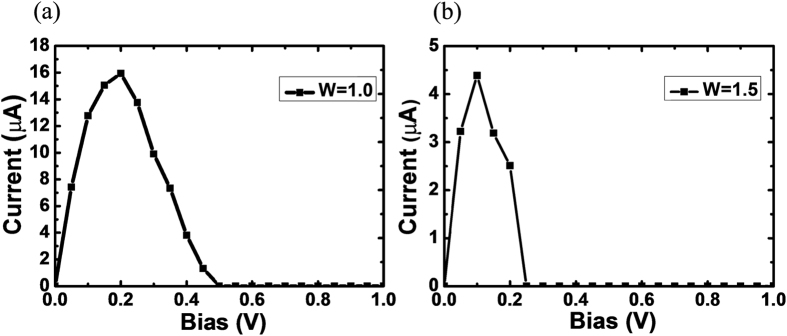
The current-voltage curve of zigzag C_2_N-*h*2D nanoribbon in the bias range from 0.0 to 1.0 V. (**a**) W = 1.0; (**b**) W = 1.5.

Figure 2(a) presents the current-voltage (*I*-*V*) characteristic of the two-probe system with W = 1.0. Apparently, a NDR peak appears in our model. The current firstly increases as the bias increases and reaches the maximum when the bias is 0.2 V and then smoothly decreases to zero as the bias increases to 0.5 V. We define 0.5 V as the cut-off bias. Interestingly, as the bias continues to increase, the current maintains at zero. It indicates that the system is in an insulator-like state. This is contrary to its metallic state under a low bias. As far as we know, this phenomenon has not been reported in periodic systems with widths of several nanometers. A similar *I*-*V* characteristic can be seen in W = 1.5 model as shown in Fig. 2(b), but the cut-off bias and the maximum current decrease due to the increased width.

**Figure 3:**
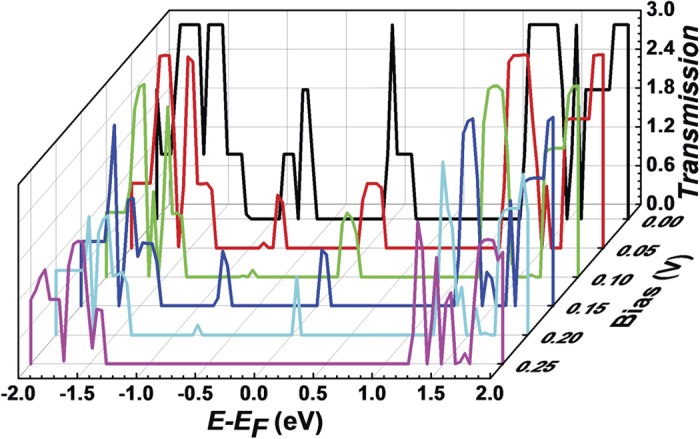
The transmission spectra of W = 1.5 zigzag C_2_N-*h*2D nanoribbon under bias *V*_*b*_ = 0.00, 0.05, 0.10, 0.15, 0.20 and 0.25 V. Zero energy is the Fermi level.

In order to investigate the physical mechanism of the peculiar *I*-*V* characteristic of our models, we first calculate the transmission spectra of W = 1.5 model under different biases as shown in Fig. 3. It can be found that as the bias increases the intensity of the transmission peak around the Fermi energy gradually decreases. Owing to the increasing integration region, the current firstly increases under a low bias. When the bias is higher than 0.1 V, the current begins to drop, so a NDR effect occurs. When the bias continues to increase to 0.25 V, the current decreases to zero due to the disappeared transmission peak. It is quite interesting that the insulator-like state can be maintained until the bias increases to 1.0 V.

**Figure 4:**
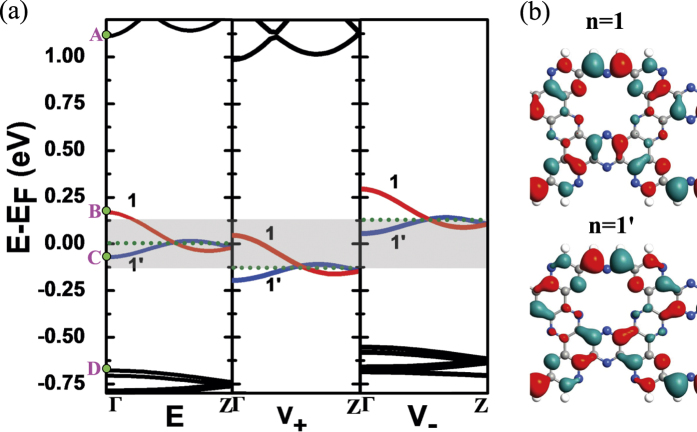
(**a**) Band structure of W = 1.5 zigzag C_2_N-*h*2D nanoribbon around the Fermi level. The left is without bias, the middle is under positive bias of 0.125 V and the right is under negative bias of −0.125 V. The two subbands near the Fermi level are shown in red (index n = 1) and blue (index n = 1′) respectively. The energy points of the four subbands near the Fermi level at the Γ-point are respectively represented by A, B, C and D. The dotted line is the Fermi level. (**b**) Isosurface plots of the Γ-point wave functions of n = 1 and n = 1′ subbands.

Figure 4(a) shows the band structure of the unit cell of the W = 1.5 zigzag C_2_N-*h*2D nanoribbon. For better illustration, we define the following variables, *E*_*AD*_ = *E*_*A*_ − *E*_*D*_, *E*_*AB*_ = *E*_*A*_ − *E*_*B*_ and *E*_*CD*_ = *E*_*C*_ − *E*_*D*_ where *E*_*A*_, *E*_*B*_, *E*_*C*_ and *E*_*D*_ are respectively the energy values of the four subbands near the Fermi level at the Γ-point. It should also be noted that *E*_*A*_ is the minimum energy value of the second band above the Fermi level, *E*_*D*_ is the maximum value of the second band below the Fermi level, *E*_*B*_ is the maximum value of n = 1 subband and *E*_*C*_ is the minimum value of n = 1′ subband. Within the energy range of the *E*_*AD*_, only two subbands cross each other and pass through the Fermi level. Both the left and right electrodes are semi-infinite zigzag C_2_N-*h*2D nanoribbons, so they also have such a band structure. If we apply a bias on the electrodes, an electric potential difference will appear between the two electrodes, and their band structures will shift downward or upward relatively. In the middle and right panels of Fig. 4(a), we applied a positive bias (*V*_+_) of 0.125 V on the left electrode and a negative bias (*V*_−_) of −0.125 V on the right electrode. As a result, there appears an energy window (the shadow region) due to the shift of the Fermi level. When the bias is low, the shadow region is small, and both n = 1 and n = 1′ subbands exist in the *V*_+_ and *V*_−_ regions, that is to say the n = 1 and n = 1′ subbands in the *V*_+_ region overlaps those in the *V*_−_ region. As the bias increases, the overlapping becomes smaller so that the transmission peak is getting lower. When the bias increases to 0.25 V, in the energy window, the n = 1 subband in the *V*_+_ region is connected to the n = 1′ subband in the *V*_−_ region. Because the electrons can not hop from one subband to another, the transmission is forbidden. Interestingly, since there are sufficient gaps both in the *E*_*AB*_ and *E*_*CD*_ ranges, the tunneling channel does not exist and the insulator-like state can be maintained as the bias continues to increase. Meanwhile, we find that the insulator-like state will happen as long as *E*_*AB*_ is greater than the absolute value of *E*_*C*_ and *E*_*CD*_ is greater than the value of *E*_*B*_. The greater *E*_*AB*_ and *E*_*CD*_ are, the greater bias range the insulator-like state can be kept in. Figure 4(b) shows the wave functions of the n = 1 and n = 1′ subbands.

**Figure 5:**
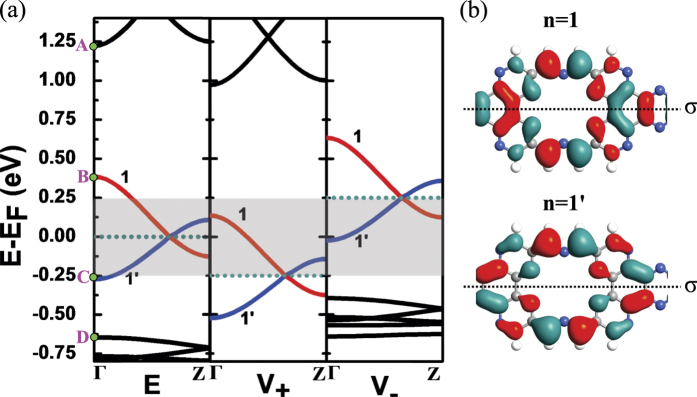
(**a**) Band structure of W = 1.0 zigzag C_2_N-*h*2D nanoribbon around the Fermi level. The left is without bias, the middle is under positive bias of 0.25 V and the right is under negative bias of −0.25 V. The two subbands near the Fermi level are shown in red (index n = 1) and blue (index n = 1′) respectively. The energy points of the four subbands near the Fermi level at the Γ-point are respectively represented by A, B, C and D. The dotted line is the Fermi level. (**b**) Isosurface plots of the Γ-point wave functions of n = 1 and n = 1′ subbands.

In Fig. 2(a), we have pointed out that the phenomenon of NDR effect and insulator-like state also occurs in W = 1.0 zigzag C_2_N-*h*2D nanoribbon, whereas the same analysis is invalid for such models with an integer width. For example, the conduction should not be cut off at 0.5 V, so this abnormal phenomenon is worthy of further investigation. Figure 5(a) shows the band structure of the unit cell of the W = 1.0 zigzag C_2_N-*h*2D nanoribbon. It is clear that W = 1.0 model has the same band morphology as W = 1.5 model, except that the n = 1 and n = 1′ subbands are more delocalized. Figure 5(b) presents the wave functions of n = 1 and n = 1′ subbands. It is easy to see that the n = 1 subband has even parity, nevertheless, the n = 1′ subband has odd parity under *σ* mirror operation. Since these two subbands have opposite *σ* parity, an electron belonging to n = 1′ subband (blue) can not hop to n = 1 (red) subband^18^,^19^, so the subbands with different colors can not couple with each other. Simultaneously, we should note that the n = 1 and n = 1′ subbands have no parity for W = 1.5 model due to its asymmetric structure as shown in Fig. 4(b), so the electrons can hop between them. This phenomenon has been reported in graphene and its related structures^19^. Under a low bias, there exists an overlapping between the subbands in the *V*_+_ and *V*_−_ region, but the electron transport only comes from the coupling between the same color subbands. When the bias increases to 0.5 V, the n = 1 subbands in the *V*_+_ and *V*_−_ region are connected in the energy window. However the n = 1 subband in the *V*_+_ region and the n = 1′ subband in the *V*_−_ region have different parity, and the tunneling between these two subbands is forbidden, so that the current is reduced to zero. This state is held as the bias continues to increase. When the bias is high, we can find the insulator-like state is still derived from the presence of band gaps in the range of *E*_*AB*_ and *E*_*CD*_. Consequently, the phenomenons of NDR effect and insulator-like state of W = 1.0 model originate from the peculiar band morphology too, except that the different parity between the subbands near the Fermi level prevents the electron transport and promotes the insulation-like state to happen in lower bias.

**Figure 6:**
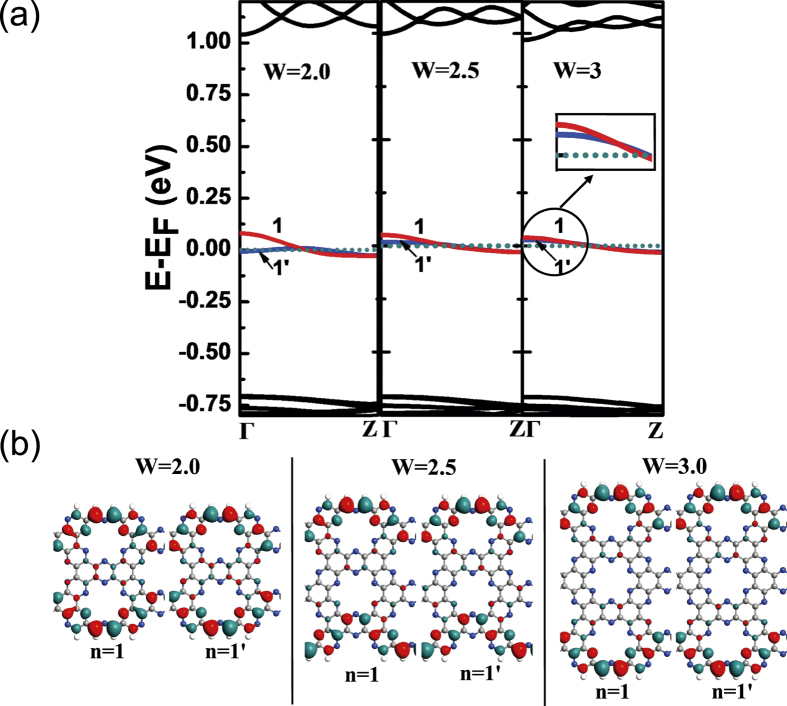
(**a**) Band structures of zigzag C_2_N-*h*2D nanoribbon around the Fermi level with different widths (W = 2.0, 2.5 and 3.0). The two subbands nearest to the Fermi level are shown in red (index n = 1) and blue (index n = 1′) respectively. The dotted line is the Fermi level. (**b**) Isosurface plots of the Γ-point wave functions of n = 1 and n = 1′ subbands.

To explore the effect of width on the electron property of zigzag C_2_N-*h*2D nanoribbon, we calculated the band structures of other models with different widths shown in Fig. 6(a). It can be seen that W = 2.0, 2.5 and 3.0 models have the same band morphology as W = 1.0 and 1.5 models, so the width does not change the main characteristics of band structure. However, as the width increases, the n = 1 and n = 1′ subbands become more localized. In Fig. 6(b), we present the wave functions of n = 1 and n = 1′ subbands. Obviously, for wider structures, the wave functions are more localized on the edge indicating the n = 1 and n = 1′ subbands are edge states which look very similar to those in zigzag graphene nanoribbons^20^,^21^. However, there is a little difference between them. In zigzag graphene nanoribbons, the localized edge states form a degenerate flat band at the Fermi level, but for our models, the subbands near the Fermi level are non-degenerated which may be caused by the introduction of N atoms^22^. And for W = 2.0 and W = 3.0 structures, the n = 1 subband has even parity and the n = 1′ subband has odd parity which is the same as W = 1.0 model. For W = 2.5 structure, n = 1 and n = 1′ subbands have no parity due to the asymmetric nanoribbon. We can conclude that as the width increases the NDR effect and the insulator-like state still exist, but the cut-off bias will become smaller due to the more localized subbands. These properties offer an interesting method by modifying the width of the zigzag C_2_N-*h*2D nanoribbon to tune the cut-off bias in future applications.

**Figure 7:**
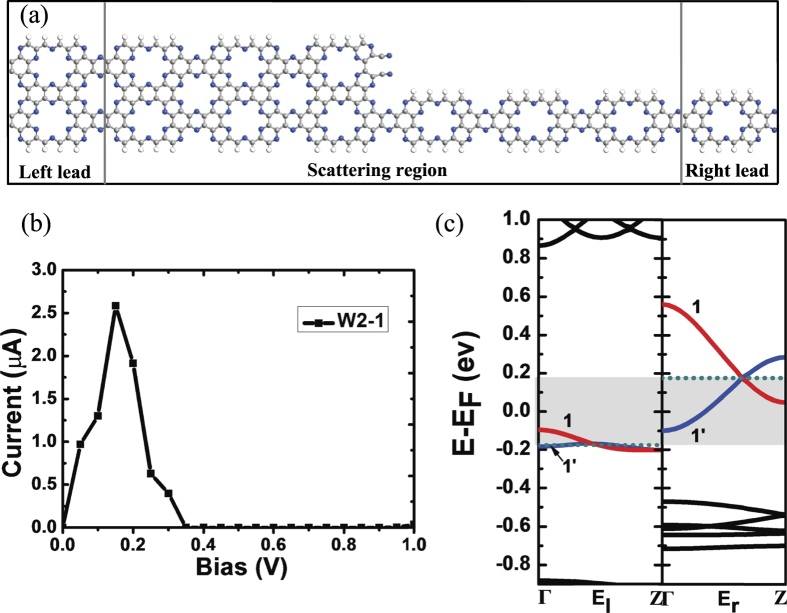
(**a**) Schematic illustration of the W2-1 model. Nitrogen, carbon and hydrogen atoms are shown by blue, gray and white balls, respectively. (**b**) The current-voltage curve of W2-1 model in the bias range from 0.0 to 1.0 V. (**c**) Band structures of the left electrode (*E*_*l*_) and rigt electrode (*E*_*r*_) of W2-1 model around the Fermi level under bias of 0.175 V and −0.175 V respectively. The two subbands nearest to the Fermi level are shown in red (index n = 1) and blue (index n = 1′) respectively. The dotted line is the Fermi level.

In the above analysis, we focus on the investigation of theoretical physical mechanism. Taking into account the possibility of experimental operation and practical application, a finite bias voltage could not be applied in a homogeneous situation with discrete translational symmetry, in order to avoid this problem we design the device of W2-1 as shown in Fig. 7(a). The central scattering region is formed by the combination of W = 2.0 and W = 1.0 nanoribbons. After optimization, some atoms at the interface of the scattering region are disturbed. To explore the influence of disturbing part on the electronic transport properties, we construct another structure W2-1-H (not shown here), two carbon atoms which were separately terminated by nitrogen atoms at the center of the scattering region are passivated by H atoms and the carbon ring has not been opened. The W2-1-H model maintains the same transport characteristics as W2-1 device. Therefore, it can be concluded that the disturbation has little effect on the whole transport properties. Figure 7(b) presents the current-voltage (*I*-*V*) characteristic of the device W2-1. It can be seen that the morphology of the *I*-*V* curve is similar to that of W = 1.0 model which is shown in Fig. 2(a). There appears an obvious negative differential resistance phenomenon. The current firstly increases as the bias increases, then begins to reduce at the bias of 0.15 V, and finally reduces to zero while the bias is 0.35 V. This insulator-like state can be maintained until the bias increases to 1.0 V. We also calculate the potential drop of the W2-1 device (not shown here), and find the potential drop mainly occurs at the center of the scattering region, so the electron scattering mainly happens around this region. In order to inspect the physics mechanism behind the phenomenon of negative differential resistance and the insulator-like state, similar to previous analysis, we calculate the band structures of the left and right electrodes of the device W2-1 in Fig. 7(c).

It can be seen that when we apply a positive bias on the device, the band structures of the left and right electrodes will shift downward or upward relatively. If the bias is low, the shadow region becomes smaller, and there will be both n = 1 and n = 1′ subbands in the *E*_*l*_ and *E*_*r*_ regions. The electrons can hop between them, so the current firstly increases. When the bias is larger than 0.15 V, n = 1 subbands do not overlap leaving only the n = 1′ subbands to contribute to the transmission, so the current starts to decrease. When the bias increases to 0.35 V, as show in Fig. 7(c), the n = 1′ subband in the *E*_*l*_ region is slightly far away from the n = 1′ subband in the *E*_*r*_ region. According to our previous analysis, the current should be zero when these two n = 1′ subbands are connected under a bias less than 0.35 V. It is noteworthy that some disturbance appears in the center of the scattering region after optimization, so the energy levels of the central part are a little different from the electrodes. That is why the current becomes zero at a higher bias. When the bias continues to increase, the current can be maintained at zero. This insulator-like state still derives from the peculiar band morphology which possesses a sufficient band gap between these two n = 1′ subbands. The physics mechanism is consistent with what we discussed previously.

## Discussion

**Figure 8:**
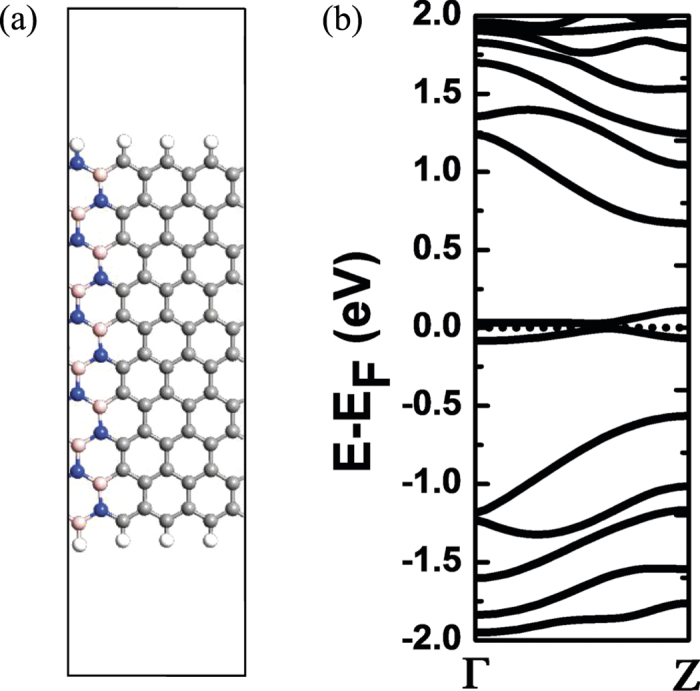
(**a**) The unit cell of C and BN hybrid zigzag nanoribbon. Nitrogen, boron, carbon and hydrogen atoms are shown by blue, pink, gray and white balls, respectively. (**b**) Band structures of C and BN hybrid zigzag nanoribbon around the Fermi level. The dotted line is the Fermi level.

In our research, we find that this mechanism can be extended to other systems, that is to say, as long as the structure has the similar band morphology, this phenomenon that the current in NDR effect can be reduced to zero and the insulator-like can be maintained will happen. For example, Kan *et al*.^23^ have reported that half-metallicity can arise in hybrid BCN nanoribbons. In Fig. 8, we give out the unit cell of the C and BN hybrid zigzag nanoribbon and its band structure. Obviously, there are only two subbands near the Fermi level, while others are far away. So it can be predicted that this phenomenon will occur in the hybrid BCN structure. Due to the limitation of the computational conditions, we do not give out the *I*-*V* curve here.

In summary, the electronic and transport properties of the zigzag C_2_N-*h*2D nanoribbons with different widths are studied using first-principles calculations. By cutting into nanoribbon, C_2_N-*h*2D changes from the original semiconductor to metallic structure owing to the introduced two edge states near the Fermi level. The band morphologies for all the nanoribbons have the same special characteristic, i.e., there are only two subbands near the Fermi level while other subbands are far away which leads to the emergence of gaps. To explore the physical mechanism, we give some homogeneous models. An obvious NDR effect and an insulator-like state occur in the *I*-*V* curve which has not been reported in such a nano-sized periodic structure in previous works. For asymmetric structures (W = 1.5 and 2.5), this phenomenon originates from the peculiar band morphology. For symmetric structures (W = 1.0, 2.0 and 3.0), besides the peculiar band morphology, the opposite parity of the two edge states is also a factor as it prevents some electron transport and promotes the insulation-like state to happen in lower bias. In addition, as the width increases, the two edge states become more localized, and the cut-off bias decreases. Therefore we can tune the electron transport by changing the width of the zigzag C_2_N-*h*2D nanoribbon. We also show a heterogeneous model composed of two zigzag C_2_N-*h*2D nanoribbons with different widths. It has a similar *I-V* curve, the peculiar band morphology still plays a decisive role and the disturbance in the center of the scattering region leads to the occurrence of the insulator-like in a little higher bias. We can also predict that for other materials, as long as they have similar band morphologies, the NDR and insulation-like state will happen.

## Methods

To investigate the transport property of our system accurately, the Atomistix Toolkit (ATK) package which is based on the combination of density functional theory (DFT) and non-equilibrium Green’s function (NEGF) technique is performed^24^. The mesh cutoff energy is set to 150 Ry, and the *k*-point mesh is 1 × 1 × 100^25^. The Perdew-Burke-Eenzerhof formulation of the generalized gradient approximation (GGA) is used as the exchange-correlation function, and the double-zeta polarized basis set is employed in the calculations. The vacuum spaces of supercell are set to be more than 15 Å to prevent the interaction with adjacent images. All the atomic positions of the structure have been optimized until all the forces are smaller than 0.01 eV/Å. The edges of zigzag C_2_N-*h*2D nanoribbon are terminated with hydrogen (H) atoms to remove the dangling bonds.

In NEGF theory, the current of the two-probe system is calculated based on the Landauer-Büttiker formula


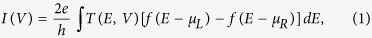


where *μ*_*L(R*)_ is the chemical potential of the left (right) electrode, *f(E* − *μ*_*L(R*)_) is the Fermi distribution of the left (right) electrode, and *V* = (*μ*_*L*_ − *μ*_*R*_)/*e* represents the bias window. *T(E, V*) is the transmission coefficient obtained by using Green’s function. Clearly, the current is determined by the area of the integral region in the bias window.

## Additional Information

**How to cite this article:** He, J.-J. *et al*. Negative differential resistance and bias-modulated metal-to-insulator transition in zigzag C_2_N-*h*2D nanoribbon. *Sci. Rep.* **7**, 43922; doi: 10.1038/srep43922 (2017).

**Publisher's note:** Springer Nature remains neutral with regard to jurisdictional claims in published maps and institutional affiliations.
